# Ischemic postconditioning ameliorates acute kidney injury induced by limb ischemia/reperfusion via transforming TLR4 and NF-κB signaling in rats

**DOI:** 10.1186/s13018-021-02565-5

**Published:** 2021-07-01

**Authors:** Zhongdi Liu, Wei Huang, Yifan Chen, Zhe Du, Fengxue Zhu, Tianbing Wang, Baoguo Jiang

**Affiliations:** National Center for Trauma Medicine, Ministry of Education Key Laboratory of Trauma and Neural Regeneration, Trauma Medicine Center, Peking University People’s Hospital, No. 11 XiZhiMen South Street, Xicheng District, Beijing, 100044 China

**Keywords:** Limb ischemia, Reperfusion injury, Kidney, Ischemic postconditioning

## Abstract

**Background:**

The present study investigated the influence of ischemic postconditioning (I-postC) on the adjustment of renal injury after limb ischemia-reperfusion (I/R) injury, to elucidate the mechanisms of the Toll-like receptor 4 (TLR 4)/NF-κB signaling pathway using histopathological and immunohistochemical methods.

**Methods:**

Male Sprague-Dawley rats were randomly assigned to five groups (numbered from 1 to 5): the sham group (Group 1, only the anesthesia procedure was conducted without limb I/R), the I/R group (Group 2, 4 h of reperfusion was conducted following 4 h limb ischemia under anesthesia), the I/R + I-postC group (Group 3, 4 h of ischemia and 4 h of reperfusion was conducted; before perfusion, 5 min of limb ischemia and 5 min of reperfusion were performed in the rats and repeated 3 times), the I/R + TAK group (Group 4, rats were injected with TLR4 antagonist TAK through the caudal vein before limb ischemia and reperfusion under anesthesia), the TAK group (Group 5, rats were injected with TAK, and the anesthesia procedure was conducted without limb I/R). Histological changes in the kidney in different groups were observed, and the extent of tubular injury was assessed. Changes in biochemical indexes and the expression of inflammatory factors, TLR4, and NF-κB were also evaluated.

**Results:**

Compared with rats in the I/R group, the secretion of inflammatory factors and the expression levels of TLR4 and NF-κB were decreased in rats in the I/R + I-postC group. Histological analysis revealed renal injury, including inflammatory cell infiltration, dilatation of the tubuli lumen, congestion in glomerular capillaries, degeneration of tubuli epithelial cells, and necrosis was ameliorated by I-postC. Immunohistochemical studies showed that I/R-induced elevation in TLR4 and NF-κB expression was reduced by I-postC treatment. Moreover, the expression levels of TLR4, NF-κB, and inflammatory factors in rats in the I/R + TAK group were also decreased, and the renal pathological lesion was alleviated, which was similar to that in rats in the I/R + I-postC group.

**Conclusions:**

The present findings suggest that I-postC can reduce tissue injury and kidney inflammation induced by limb I/R injury, possibly via inhibition of the TLR4 and NF-κB pathways.

## Background

Ischemia-reperfusion (I/R) injury is a common pathological state, which is characterized by the restriction of blood supply to organs or tissues, followed by the recovery of blood perfusion and oxygen supply [1, 2]. However, the recovery of blood perfusion and oxygen supply leads to severe inflammatory reactions and aggravation of tissue injury [3, 4]. Limb I/R often occurs in the field of trauma surgery and has a wide range of effects [5, 6]. Short-term ischemia of skeletal muscle can cause irreversible damage, which not only affects the survival and function of ischemic limbs, but may also cause systemic inflammatory response syndrome, leading to multiple organ failure [7, 8]. Previous studies have shown that ischemia and subsequent reperfusion could trigger local and systemic damage with the involvement of free oxygen radicals and inflammatory mediators [9]. Although blood flow protects the extremities from necrosis, multi-organ dysfunction may progress and even result in death [10, 11].

The kidney is highly sensitive to ischemia and hypoxia, and is prone to acute injury in the process of limb I/R injury, which is characterized by blurred swelling in the tubuli epithelium, acute tubular necrosis, inflammatory cellular infiltration, edema, and congestion [12, 13]. Acute kidney injury induced by limb I/R involves complex mechanisms, and inflammatory reactions and oxidative stress play an important role in this pathological process [11, 14, 15]. Some characteristic endogenous proteins are produced in the early phase, and previous studies have shown that the Toll-like receptor 4 (TLR4) signaling pathway is closely related to inflammatory response [16]. Activation of TLR4 can promote the translocation and expression of inflammatory mediators such as NF-κB, resulting in an increased release of downstream inflammatory molecules, including interleukin-6 (IL-6), IL-10, and tumor necrosis factor-α (TNF-α) [17]. Thus, the TLR4/NF-κB signaling pathway may be involved in renal injury after limb I/R.

Currently, many treatment procedures and medications for preventing and ameliorating kidney injury induced by limb I/R are being developed [18, 19]. Ischemic postconditioning (I-postC) is a promising method to alleviate remote organ dysfunction by performing several cycles of transient ischemia and reperfusion intervention on the ischemic limb. Many studies have shown that I-postC could reduce the inflammatory response and protect organ function via several pathways during I/R injury of the heart, lung, liver, and other tissues [20–22]. However, whether the TLR4/NF-κB pathway is involved in the process of kidney injury induced by limb I/R and related mechanisms is not clear. In this study, we established a limb I/R injury rat model and evaluated the protective effect of I-postC on remote kidney function. Additionally, the role of TLR4/NF-κB signaling in the renal protective effects of I-postC after limb I/R was further explored.

## Methods

### Animals

Male Sprague-Dawley rats, aged 8–10 weeks and weighing 300–350 g, were acquired from The Animal Center of Peking University People’s Hospital. All experimental animals were specific-pathogen-free and housed in separate cages, with a stable temperature and relative humidity, and had free access to food and water until 12 h before the experiment. All animal experiments were conducted in accordance with the guidelines of the National Institutes of Health for the Care and Use of Laboratory Animals and approved by The Animal Research Ethics Committee of Peking University People’s Hospital (Approval number: 2020PHE067).

### Establishment of rat model with limb I/R injury and experiment design

**Fig. 1:**
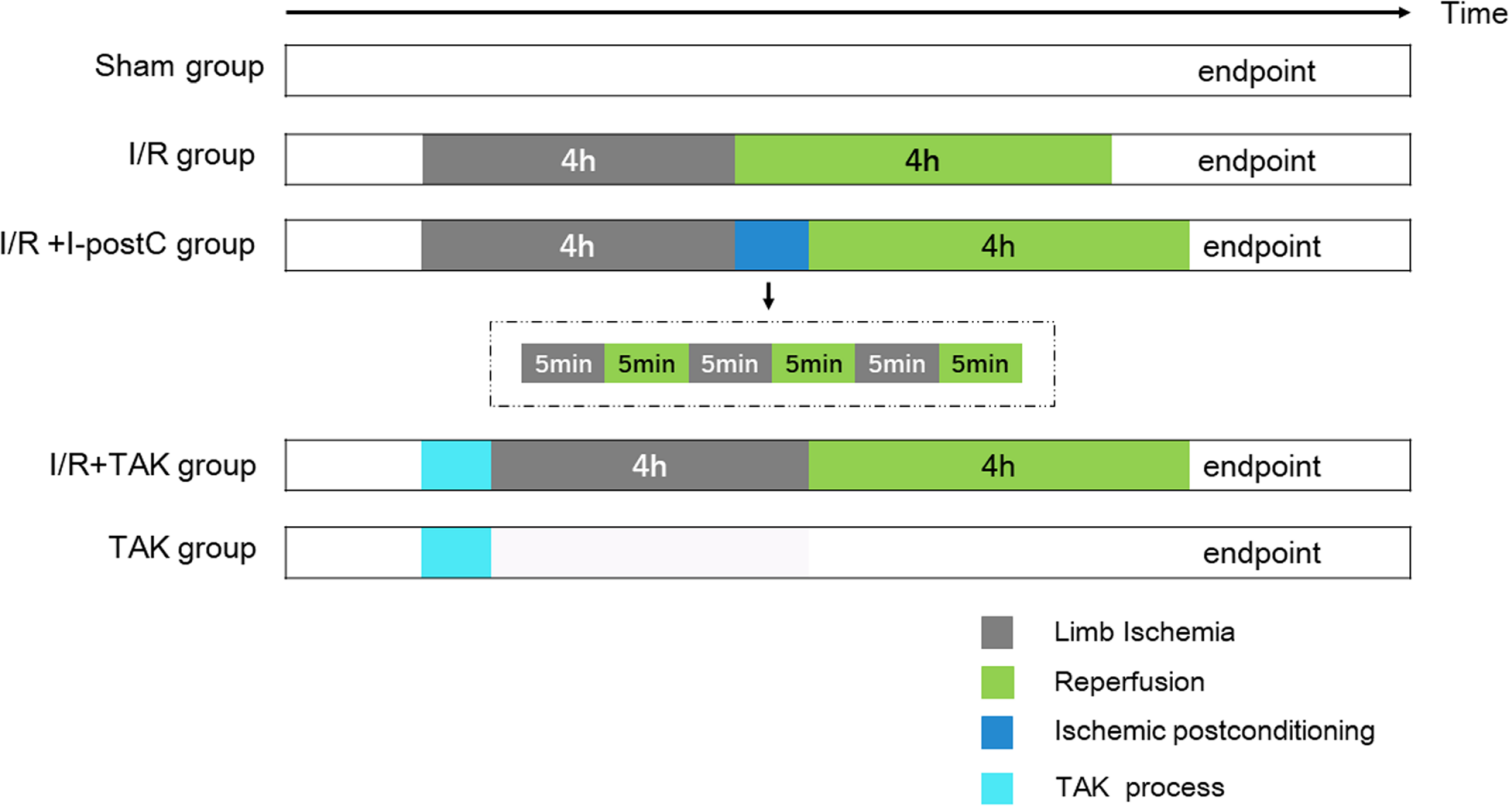
Schematic illustration of the experimental protocols. * I/R*, ischemia-reperfusion; *I–postC*, ischemic postconditioning

The rats (*n* = 30) were randomly divided into five groups (numbered from 1 to 5, *n* = 6 in each group). All rats were anesthetized by Zoletil 50 injection (40 mg/kg, intraperitoneally [i.p.]), and a warming table with constant temperature was used to maintain body temperature. In the I/R group (Group 2), the blood flow of the right hind limb was blocked by a self-locking nylon band (width 5 mm) above the trochanter to block the arterial blood for 4 h, followed by reperfusion for 4 h. The blocking pressure was monitored by a pressure monitoring device and maintained at 300 ± 20 mmHg. When the paw of the rat's toes became pale, blue, and cool, and the laser Doppler blood flow imaging instrument verified that the blood flow of the hind limb was successfully blocked, the model was established successfully. No drugs or I-postC was administered. At the end of the experiment, the rats were sacrificed by piercing the inferior vena cava for blood collection until cessation of breathing and the heartbeat. In the sham group (Group 1), the band was put in place without being fastened, and the anesthesia procedure was conducted. In the I/R + I-postC group (Group 3), the blood flow of the right hind limb was blocked for 4 h, followed by 5 min ischemia and 5 min reperfusion intervention for 3 cycles (30 min in total), and followed by 4 h of reperfusion. Rats in the I/R + TAK group (Group 4) were injected with TAK-242 (TLR4 antagonist, 1.5 mg/kg, i.p.) before 4 h of limb ischemia and 4 h of reperfusion under anesthesia. Rats were injected with TAK-242 (1.5 mg/kg, i.p.), and the anesthesia procedure was conducted without limb I/R in the TAK group (Group 5). Rats in other groups were injected with the same volume of normal saline. In this study, all rats survived the establishment of the model, and blood and kidney tissues were collected under anesthesia after the operations. The experimental schedule is presented in Fig. 1.

### Kidney tissue wet/dry weight ratio and renal function analysis

The right upper renal tissue was immediately weighed (wet weight) after excision. The excised tissues were dried at 60 °C for 2 days to obtain a stable dry weight, before the wet/dry weight ratio was calculated. Blood samples were collected from the inferior vena cava and centrifuged at 3000 rpm for 10 min. The supernatant of each sample was collected to evaluate blood urea nitrogen (BUN), serum creatinine (SCr), and creatine kinase (CK) using clinically automated analysis methods (Rayto, Chemray 800).

### Histological analysis

The kidney samples were collected and perfused with cold saline and 4% paraformaldehyde. After fixation for 24 h at 4 °C, the tissues were embedded in paraffin and prepared into 5-μm sections, before being stained with hematoxylin and eosin (H&E) for 5 min at room temperature. Then, the kidney sections were observed under a standard light microscope (ECLIPSE E100, NIKON, Japan) by an experienced pathologist who was blinded to the content of this study. All microscopic assessments of the sections were performed in a randomized manner. The key points of interest of the pathological observation of the renal tubular injury included the degree of tubular dilatation, casting formation, tubular vacuolization, and necrosis.

### Measurement of IL- 6, IL-10, and TNF-α concentrations

The concentrations of IL-6, IL-10, and TNF-α in the rat serum and kidney were measured using ELISA kits according to the manufacturer's instructions (MultiSciences Biotech Co., Ltd. and Thermo Fisher Scientific Inc.). The catalog numbers for the ELISA kits were IL-6 (EK306/3-48), IL-10 (EK310/2-48), and TNF-α (88-7340). The absorbance value at 450 nm was measured by a multifunctional microplate reader (BioTek Epoch, USA).

### Immunohistochemical analysis

TLR4 and NF-κB p65 expression in kidney tissues was evaluated using immunohistochemistry. Tissue sections (5 μm) were prepared according to the above steps and heated in an oven for 1 h, dewaxed in xylene, rehydrated using graded ethanol solutions, and microwaved at 100 °C in sodium citrate buffer for 20 min. The slides were then cooled to room temperature and incubated in 3% H_2_O_2_ for 10 min. The sections were blocked using bovine serum albumin at room temperature for 30 min and then incubated at 4 °C overnight with a primary antibody against TLR4 (1:100; cat. no. AF7017; Cell Signaling Technology) and primary antibody against NF-κB (1:100; cat. no. #38054; Cell Signaling Technology). Then, samples were washed with phosphate-buffered saline three times for 5 min each, before incubation with goat anti-mouse secondary antibody (1:200. cat. no. GB23301; Servicebio) labeled with diaminobenzidine (DAB; no. G1211; Servicebio). The samples were then incubated with 3,3'-diaminobenzidine substrate for 30 s at room temperature. After dehydration and drying, the sections were embedded with neutral glue and observed by light microscope (magnification, ×200). Immunohistochemical observations were evaluated with Image Pro Plus software. The average optical density was used to evaluate TLR4 and NF-κB p65 expression and compared between groups.

### Western blot analysis

The frozen renal tissue samples were dissolved in RIPA buffer (cat. no. G2002, Servicebio) on ice by a homogenizer. Then the samples (50 μg/lane) were separated on a sodium dodecyl sulfate-polyacrylamide gel (12% gel) and electrotransferred onto polyvinylidene fluoride membranes. After blocking in 5% non-fat milk for 1 h at room temperature, the membranes were incubated with primary antibodies on a table concentrator overnight at 4 °C. Primary antibodies against β-actin (1:1000; cat. no. GB12001; Servicebio), TLR4 (1:1000; cat. no. GB11519; Servicebio), and NF-κB p65 (1:2000; cat. no. GB11142; Servicebio) were used. After washing the membranes with Tris-buffered saline Tween (TBST) three times, a horseradish peroxidase-conjugated secondary antibody (1:5000; Servicebio) was added at room temperature for 1 h. The membranes were subsequently washed three times with TBST and visualized by electrochemiluminescence. The images were analyzed with the Image Lab Analysis System (Alpha Innotech, alphaEaseFC; Adobe, Adobe PhotoShop).

### Statistical analysis

All data are presented as mean ± SD and analyzed by one-way ANOVA. If the analysis of variance revealed a significant difference, then Tukey's post hoc test was performed for pairwise comparison among groups. The level of statistical significance for all analyses was set at p < 0.05. Analysis was performed using SPSS version 19.0 software (IBM SPSS Statistics).

## Results

### Limb ischemia-reperfusion injury caused morphological and functional impairment of the kidneys in rats

**Fig. 2.**
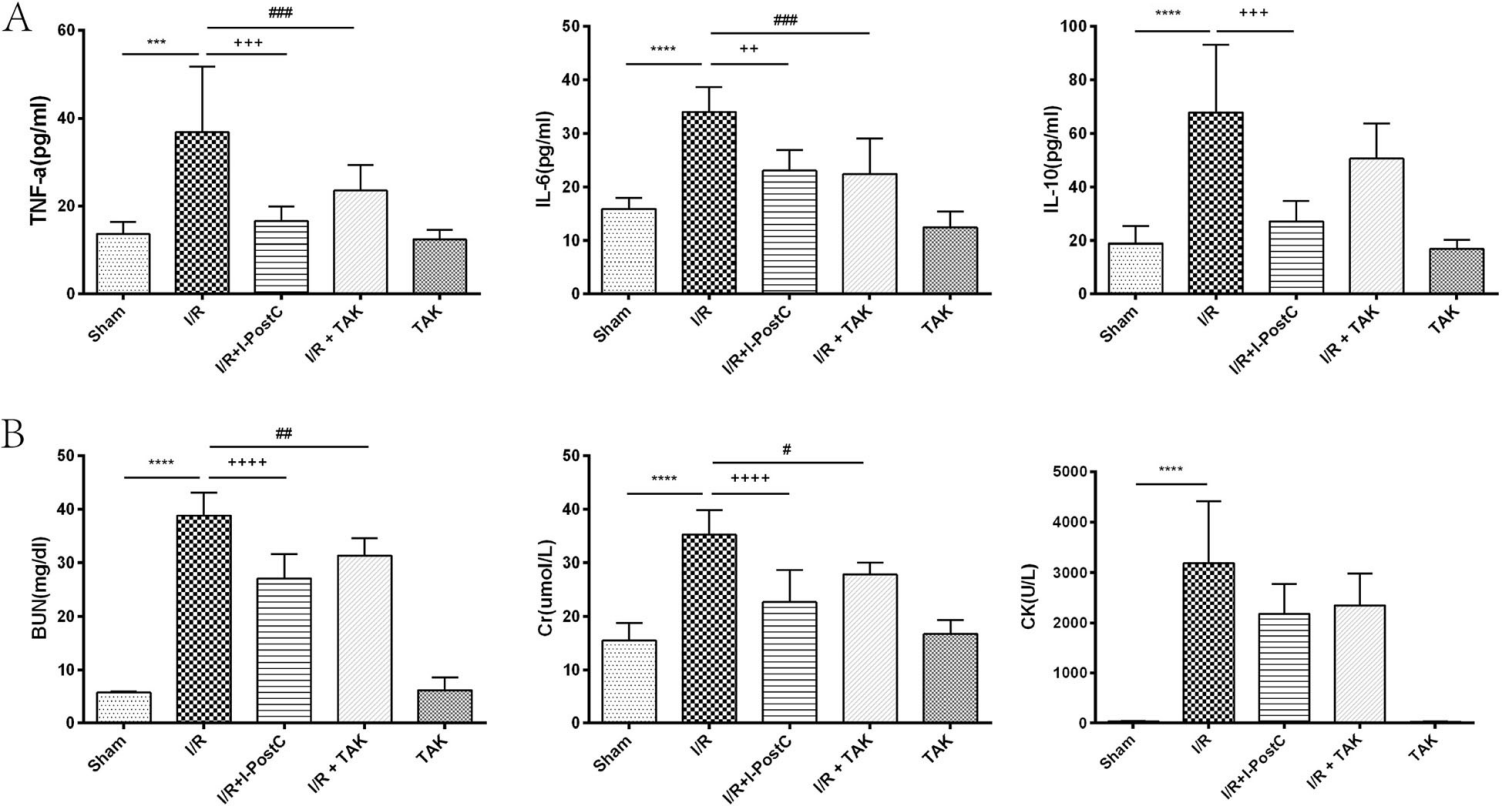
Serum levels of **A** TNF-α, IL-6, and IL-10, and **B** BUN, Cr, and CK, in rats from different groups. Data are presented as mean ± SD. N = 6 in each group. *** *P* < 0.001, **** *P* < 0.0001, I/R vs. Sham; ++ *P* < 0.01, +++ *P* < 0.001, ++++ *P* < 0.00001, I/R + I-postC vs. I/R; # *P* < 0.05, ## *P* < 0.01, ### *P* < 0.001, I/R + TAK vs. I/R. *Cr*, creatinine; *BUN*, blood urine nitrogen; *CK*, creatine kinase

**Fig. 3:**
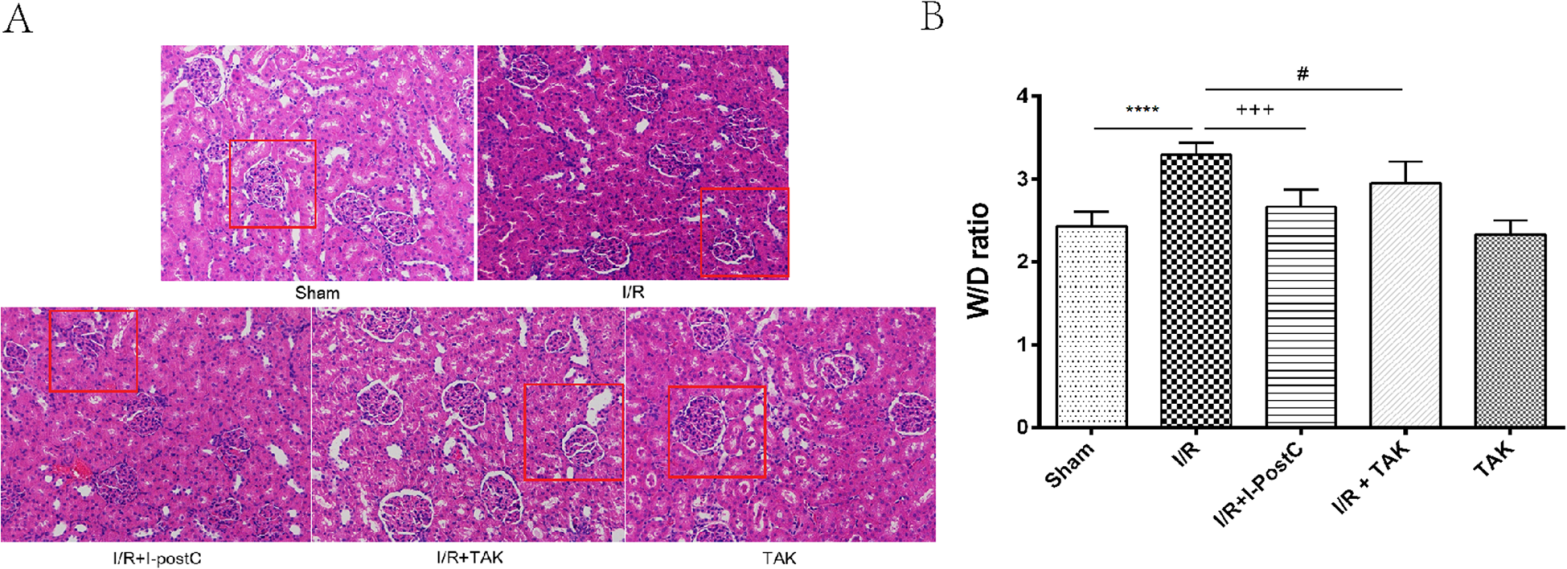
Renal morphologic features evaluated by H&E staining (×200) and W/D ratio in different groups. **A** Sham, normal glomeruli and tubules; I/R, diffuse glomerular atrophy, renal tubular epithelial cell edema, lumen stenosis, and occlusion; I/R + I-postC, segmental atrophy of glomerulus with slight edema of tubular epithelial cells; I/R + TAK, segmental glomerular atrophy with mild to moderate tubular stenosis; TAK, no significant changes in the morphology of glomeruli and renal tubules. **B** **** *P* < 0.0001, I/R vs. Sham; +++ *P* < 0.001, I/R + I-postC vs. I/R; # *P* < 0.05, I/R + TAK vs. I/R

First, we examined the effectiveness of the animal model of renal injury induced by limb I/R in rats. Compared with rats in Group 1, the levels of Cr, BUN, and CK in the serum of rats in Group 2 were significantly increased after 4 h of ischemia and 4 h of reperfusion, as well as the expression levels of TNF-α, IL-6, and IL-10; edema of the kidney interstitium and destruction of the renal tubular epithelial cells were also observed. The wet/dry weight ratio was significantly higher in rats in Group 2 than in rats in Group 1. These results demonstrated that the animal model of I/R-induced renal injury had been successfully established (Figs. 2 and 3).

### I-postC alleviates kidney injury induced by limb I/R in rats

We evaluated the specific effect of I-postC on renal injury after limb I/R by measuring the levels of serum inflammatory cytokines and biochemical indexes. Compared with rats in Group 2, the levels of IL-6, IL-10, and TNF-α in the serum were significantly inhibited in rats in Group 3, and the degree of pathological injury of the kidney tissue was also significantly reduced. At the same time, we observed that the levels of inflammatory cytokines were also inhibited in rats in Group 4, and kidney function and histopathological changes were improved, as in Group 3. The wet/dry weight ratio was significantly reduced in rats in both Group 4 and Group 3, compared with rats in Group 2. The levels of all indexes in rats in Group 5 were similar to those in rats in Group 1 (Figs. 2 and 3). These results demonstrated that I-postC could exert a protective effect against limb I/R-mediated kidney injury by regulating the secretion of inflammatory cytokines in rats.

### I-postC inhibits activation of TLR4/NF-κB signaling in rats with limb I/R injury

**Fig. 4:**
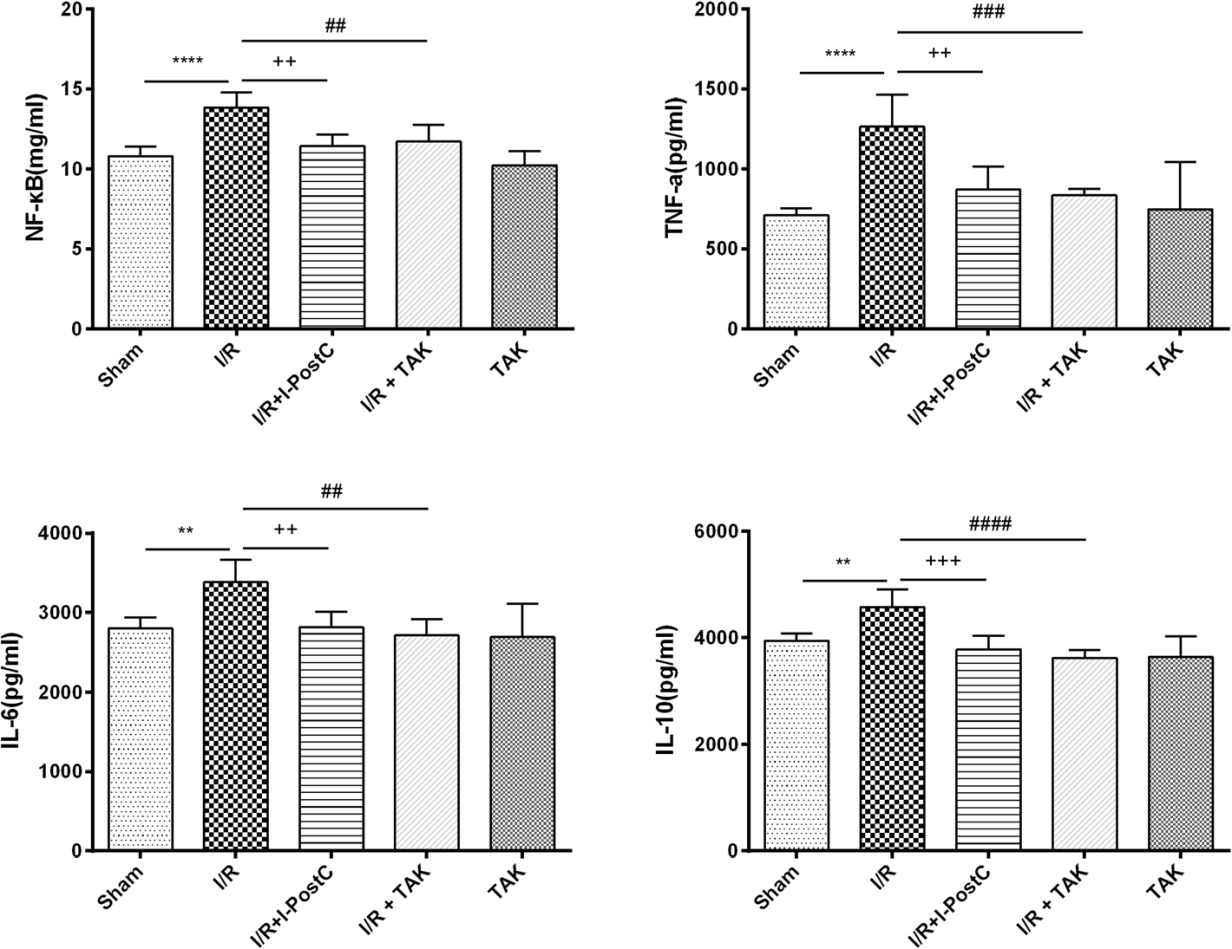
NF-κB, TNF-α, IL-6, and IL-10 levels in the kidneys of rats in different groups. Data are presented as the mean ± SD. N = 6 in each group. ** *P* < 0.01, **** *P* < 0.0001, I/R vs. Sham; ++ *P* < 0.01, +++ *P* < 0.001, I/R + I-postC vs. I/R; ## *P* < 0.01, ### *P* < 0.001, #### *P* < 0.0001, I/R + TAK vs. I/R. TNF-α, tumor necrosis factor-α; *IL*, interleukin

Since TAK exhibits a nephroprotective effect, we concluded that the regulation of TLR4 levels may play an important role in the remission of I/R-induced renal injury. Thus, the effect of I-postC on the TLR4/NF-κB signaling pathway was examined. The levels of inflammatory compounds in the renal tissues, including NF-κB, IL-6, IL-10, and TNF-α, were measured to validate the protective effect of I-postC on renal inflammation after limb I/R. The secretion of these factors was significantly increased in rats in Group 2 compared with that in sham rats. However, I-postC or TAK processing effectively inhibited limb I/R-induced secretion of the inflammatory cytokines NF-κB, TNF-α, IL-6, and IL-10 (Fig. 4).

**Fig. 5:**
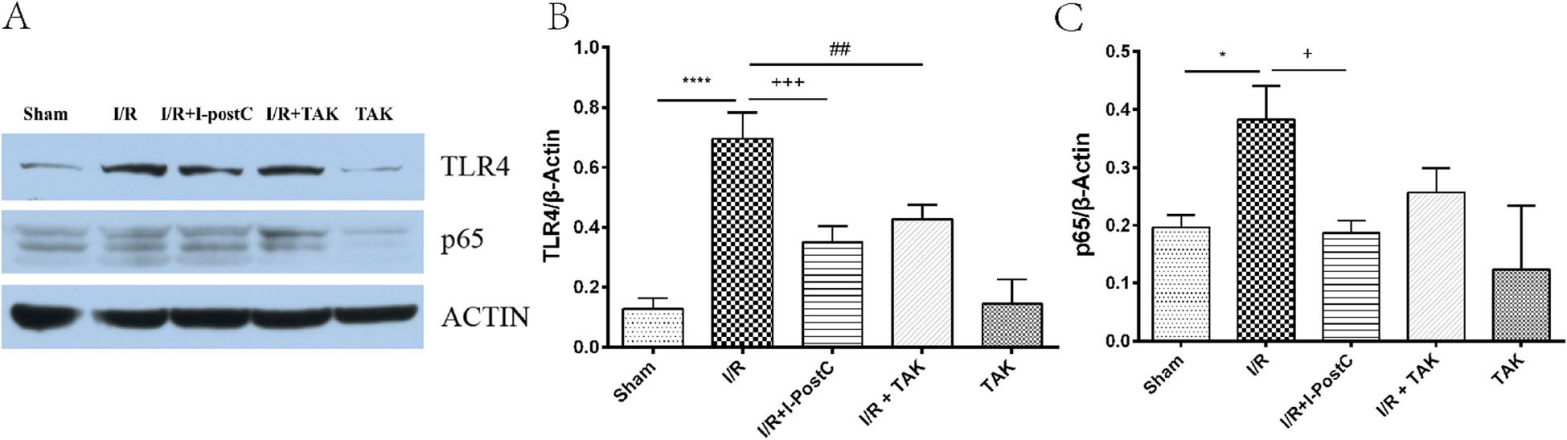
Representative western blotting images (**A**) and statistical analysis of TLR4 (**B**) and NF-κB p65 (**C**) expression levels in the kidneys of rats in different groups. Data are presented as the mean ± SD. *N* = 6 in each group. * *P* < 0.05, **** *P* < 0.0001, I/R vs. Sham; + *P* < 0.05, +++ *P* < 0.001, I/R + I-postC vs. I/R; ## *P* < 0.01, I/R + TAK vs. I/R. *TLR4*, toll-like receptor 4

**Fig. 6:**
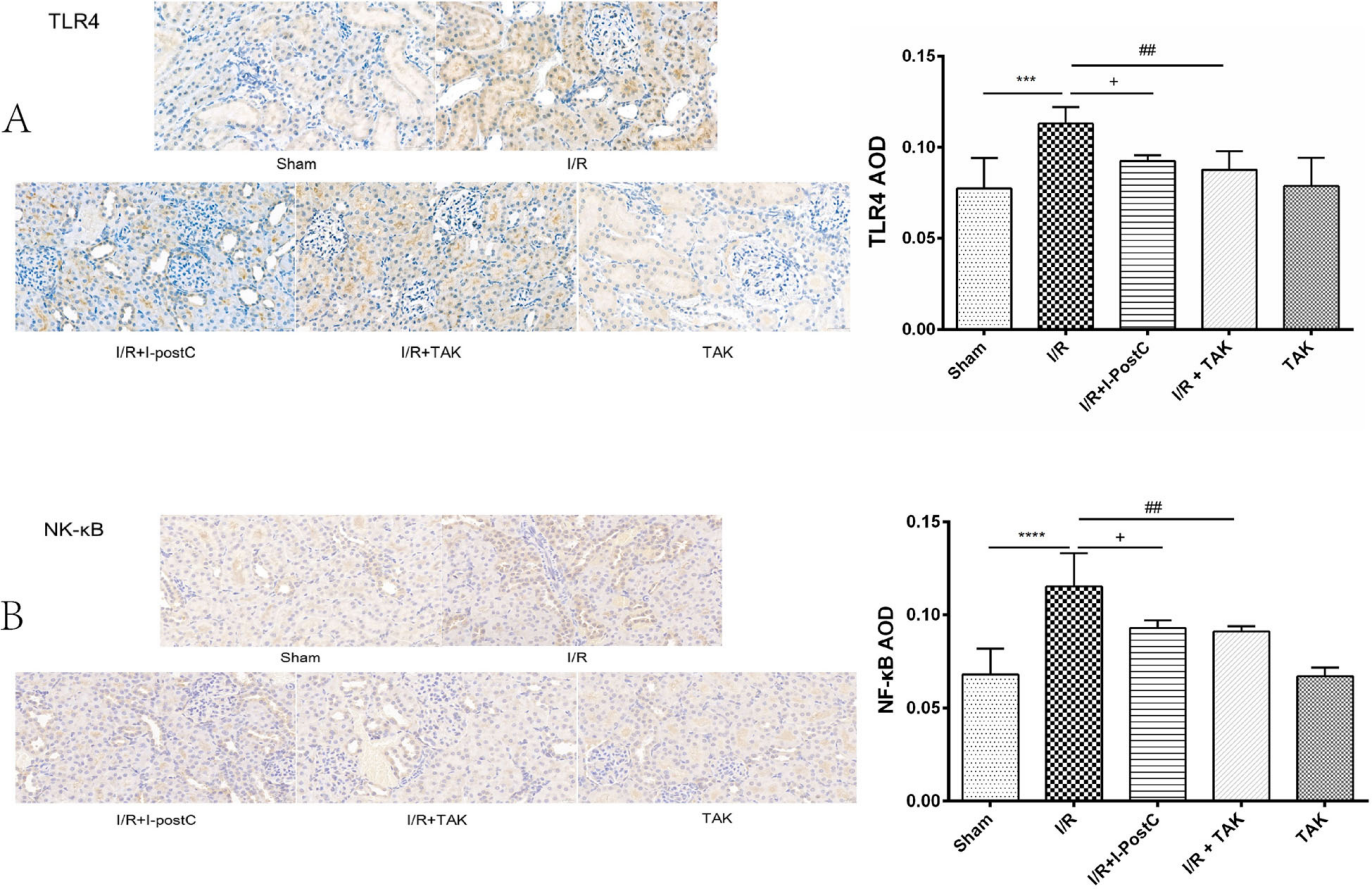
Representative immunohistochemistry images and statistical analysis of **A** TLR4 and **B** NF-κB p65 expression levels in the kidneys of rats in different groups. Data are presented as the mean ± SD. N = 6 in each group. Scale bar, 20 μm. *AOD*, average optical density

Meanwhile, we detected the expression levels of TLR4 and NF-κB in the kidney of rats in different groups by western blot and immunohistochemical analysis. The result of western blot analysis showed that the secretion of TLR4 was significantly increased in the renal tissue of rats in Group 2 compared with that of rats in Group 1, and were effectively inhibited in Group 3, which was consistent with the result of immunohistochemical analysis. Similarly, both western blot and immunohistochemical analysis demonstrated that NF-κB expression was increased in rats in Group 2, compared with that in rats in Group 1, and were significantly reduced in rats in Group 3 (Figs. 5 and 6). These results suggested that I-PostC intervention could effectively inhibit the activation of TLR4/NF-κB signaling and alleviate kidney injury induced by limb I/R in rats.

## Discussion

Limb I/R injury is a common type of injury in the field of trauma surgery, and is prevalent during earthquakes, mining accidents, traffic accidents, and other disasters. The rate of death and disability is very high; previous studies have shown that a variety of pathophysiological processes are involved in this injury [23]. Limb I/R includes two classic processes, namely tissue ischemia and reperfusion injury. Ischemic injury is mainly caused by hypoxia of tissue cells, which leads to cell swelling, degeneration, and even necrosis [24]. Reperfusion injury is mainly caused by inflammatory factors and mediators, including oxygen free radicals, neutrophils, and nitric oxide [25].

After limb ischemia, skeletal muscle metabolism changes from aerobic to anaerobic, resulting in acidosis caused by a large amount of lactic acid accumulation in the tissue, and skeletal muscle injury gradually worsens, eventually leading to severe destruction of skeletal muscle cell structure, inflammatory cell infiltration, and muscle cell necrosis [6, 8]. In addition, muscle tissue cytokines are released, white blood cells are activated, the expression of cell adhesion molecules is increased, toxic oxygen metabolites and inflammatory mediators begin to enter the circulatory system causing kidney, liver, lung, and other distant organ tissue damage, leading to dysfunction of multiple organ systems [7]. However, the recognition of remote organ injury after limb I/R is far from sufficient, and many aspects of its pathogenesis remain unclear, which warrants further study.

In recent years, research on the protection of organ injury after limb I/R has been widely studied in clinical practice. I-postC is an important endogenous protective measure to alleviate I/R-related injury. It refers to the repetitive application of ischemia and reperfusion treatment before ischemic tissue and organ recover blood supply, which can effectively improve the degree of I/R injury in tissues and organs. I-postC was initially applied for cardiac protection during a canine myocardial infarction model experiment [20]. It has been confirmed that I-postC plays a protective role in I/R injury of the heart, kidney, brain, liver, and other organs [22, 26]. I-postC can effectively reduce renal tissue apoptosis caused by I/R injury, prevent renal insufficiency, and reduce the release of inflammatory factors after I/R. It can also inhibit renal fibrosis and improve the function of the transplanted kidney [27]. However, the effect of I-postC on renal injury induced by limb I/R has rarely been studied, and the specific mechanism has not been fully clarified. In the present study, we established a rat model of limb I/R injury to determine whether I-postC can prevent or reduce distal kidney injury caused by limb I/R, and to explore its mechanism.

The TLR4/NF-κB signaling pathway is a signal transduction pathway closely related to the anti-inflammatory immune mechanism, which plays an important role in the occurrence and development of tissue inflammation [16, 28]. TLR4 is a recognition receptor on the surface of immune cells that identifies pathogen-related molecules, and plays an important role in the activation of signal transduction. It is widely expressed in a variety of human cells, mainly in monocytes/macrophages, vascular endothelial cells and renal tubular epithelial cells [17, 29]. NF-κB is widely expressed in eukaryotic cells, and is mainly involved in intracellular information transfer and the expression of related inflammatory factors. It is largely involved in signal transduction, immune regulation, stress response, and apoptosis, and plays an important role in the initiation of inflammatory cascade reactions [30]. Activation of TLR4/NF-κB signaling can regulate gene transcription and the expression of many pro-inflammatory mediators related to immunity and inflammation, as well as regulate the gene transcription process of a variety of cytokines and adhesion molecules involved in I/R injury, so as to control their biosynthesis, which plays an important role in the occurrence and development of limb I/R injury. For example, it can effectively induce the gene expression of a variety of cytokines (such as IL-6 and TNF-a), adhesion molecules (such as ICAM-1 and VCAM-1), and chemokines (such as COX-2). TLR4/NF-κB can regulate inflammation upstream, thus becoming the hub and key factor of inflammation regulation [31]. Moderate intervention of TLR4/NF-κB activation is of great clinical value.

IL-6 is a versatile pleiotropic cytokine that regulates immune response, acute phase response, and hematopoiesis, and plays an important role in the body’s anti-infection immune response. When infection and inflammation occur, IL-6 is produced by monocytes and macrophages stimulated by Toll-like receptors (e.g., TLR 4) and its level rapidly increases, the magnitude of the increase reflects the severity of the disease [32, 33]. IL-10 is a multicellular and multifunctional cytokine, which regulates cell growth and differentiation, participates in inflammatory and immune responses, and is recognized as an anti-inflammatory and immunosuppressive factor [34, 35]. IL-10 expression is strictly regulated and is often accompanied by the expression of pro-inflammatory cytokines. Previous studies have shown that NF-κB activation downstream of pattern recognition receptor could regulate IL-10 production in myeloid cells, and several NF-κB family members could promote IL-10 production in TLR4-stimulated macrophages [36]. In this study, IL-6 and IL-10 levels in plasma and renal tissues were both significantly increased after limb I/R, suggesting that acute injury activated both pro-inflammatory and anti-inflammatory responses in the body. The level of IL-10 was significantly decreased while the level of IL-6 was decreased by I-postC, both in plasma and renal tissues, together with TLR 4 and NF-κB expression levels. These results suggest that I-postC could reduce the expression of both pro-inflammatory and anti-inflammatory factors through possible regulation of the TLR4/NF-κB signaling pathway and play a role in renal protection.

In addition, we compared results between rats in the I/R + TAK and I/R groups, which showed limited activation of the TLR4/NF-κB pathway, and the expression level of inflammatory factors was significantly decreased, showing similar protective effects as I-postC. Thus, we believe that I-postC could effectively reduce the inflammatory reaction in the whole body and distant organs after limb I/R by transforming the TLR4/NF-κB pathway and inhibiting the accumulation of inflammatory cells in the tissues. However, the process of limb I/R causing distant organ injury is very complex, and many pathophysiological processes are involved [3, 6, 9]. Although we explored the possible role of TLR4/NF-κB pathway in this process, it is still necessary to acknowledge that multiple regulatory mechanisms simultaneously in this process, which requires further investigation.

There are still some limitations in our study. First, the observation period was relatively short. It is not clear whether I-postC has a long-term protective effect on renal injury and the related mechanism. Second, the animals used in this study were all male animals. Although the impact of hormone level fluctuations on the test data may be avoided, the single-sex animals may cause certain bias in the results. Therefore, in further studies, we will improve the experimental design and explore the long-term effect and mechanism of I-postC on the protection of distant organs.

## Conclusions

In conclusion, this study confirmed that the TLR4/NF-κB signaling pathway is involved in kidney injury after limb I/R. Limb I/R can activate the TLR4/NF-κB signaling pathway within the kidneys to produce an inflammatory response, which leads to renal injury, while inhibition of the signal pathway can reduce the release of inflammatory factors and the degree of renal injury. I-postC can significantly reduce the degree of renal injury after limb I/R by inhibiting the release of inflammatory factors mediated by TLR4/NF-κB signaling. Although research into I-postC in the prevention and treatment of kidney injury induced by limb I/R is still in the fundamental stages, I-postC exhibits post-traumatic, noninvasive, and exact curative effects, and has broad application prospects.

## Data Availability

The datasets are available from the corresponding author on reasonable request.

## Abbreviations

ANOVA: Analysis of variance
BUN: Blood urea nitrogen
CK: Creatine kinase
H&E: Hematoxylin and eosin
I/R: Ischemia-reperfusion
IL: Interleukin
I-postC: Ischemic postconditioning
SCr: Serum creatinine
TBST: Tris-buffered saline Tween
TLR 4: Toll-like receptor 4
TNF-α: Tumor necrosis factor-α
